# Autoantibodies to Killer Cell Immunoglobulin-Like Receptors in Patients With Systemic Lupus Erythematosus Induce Natural Killer Cell Hyporesponsiveness

**DOI:** 10.3389/fimmu.2019.02164

**Published:** 2019-09-11

**Authors:** Filip Segerberg, Christian Lundtoft, Sarah Reid, Karin Hjorton, Dag Leonard, Gunnel Nordmark, Mattias Carlsten, Niklas Hagberg

**Affiliations:** ^1^Department of Medicine, Center for Haematology and Regenerative Medicine, Karolinska Institutet, Stockholm, Sweden; ^2^Rheumatology and Science for Life Laboratories, Department of Medical Sciences, Uppsala University, Uppsala, Sweden

**Keywords:** autoantibody, killer cell immunoglobulin-like receptor, systemic lupus erythematosus, nephritis, natural killer cells, primary Sjögren's syndrome

## Abstract

Natural killer (NK) cell cytotoxicity toward self-cells is restrained by the inhibitory HLA class I-binding receptors CD94/NKG2A and the killer cell immunoglobulin-like receptors (KIRs). CD94/NKG2A and KIRs are also essential for NK cell education, which is a dynamic functional maturation process where a constitutive binding of inhibitory receptors to cognate HLA class I molecules is required for NK cells to maintain their full cytotoxic capacity. Previously, we described autoantibodies to CD94/NKG2A in patients with systemic lupus erythematosus (SLE). In this study we analyzed sera from 191 patients with SLE, 119 patients with primary Sjögren's syndrome (pSS), 48 patients with systemic sclerosis (SSc), and 100 healthy donors (HD) for autoantibodies to eight different KIRs. Anti-KIR autoantibodies were identified in sera from 23.0% of patients with SLE, 10.9% of patients with pSS, 12.5% of patients with SSc, and 3.0% of HD. IgG from anti-KIR-positive SLE patients reduced the degranulation and cytotoxicity of NK cells toward K562 tumor cells. The presence of anti-KIR-autoantibodies reacting with >3 KIRs was associated with an increased disease activity (*p* < 0.0001), elevated serum levels of IFN-α (*p* < 0.0001), nephritis (*p* = 0.001), and the presence of anti-Sm (*p* = 0.007), and anti-RNP (*p* = 0.003) autoantibodies in serum. Together these findings suggest that anti-KIR autoantibodies may contribute to the reduced function of NK cells in SLE patients, and that a defective NK cell function may be a risk factor for the development of lupus nephritis.

## Introduction

Natural killer (NK) cells are cytotoxic cells that can kill virally infected and tumor transformed cells as well as activated immune cells. To discriminate target cells from healthy self-cells, NK cells are equipped with a large array of inhibitory and activating receptors, which sense the expression of HLA class I molecules and stress-induced ligands, respectively (1). The inhibitory receptors primarily consist of the CD94/NKG2A receptor and the killer cell immunoglobulin-like receptor (KIR) family.

The KIR family is encoded by 17 genes, including two pseudogenes, on chromosome 19 (2). Each receptor is named based on the number of extracellular immunoglobulin-like domains (KIR2D or KIR3D), the presence of a long (L) or a short (S) cytoplasmic tail, and a digit (1–5) for discrimination of subtypes within these groups. Generally, KIRs with two extracellular domains bind HLA-C allotypes, whereas KIRs with three extracellular domains bind HLA-A, or HLA-B allotypes. The long cytoplasmic tail contains ITIM motifs and therefore act as inhibitory receptors, whereas the short cytoplasmic tail associates with the adaptor protein DNAX activation protein 12 (DAP12) that contain ITAM motifs (3), which deliver an activation signal. The number and types of KIRs expressed varies between individuals, but the *KIR* gene content can broadly be defined by two haplotypes. The A haplotype mainly encode a fixed set of inhibitory and one activating receptor, whereas the B haplotype has a variable number of inhibitory, and several activating receptors (4).

In addition to restraining NK cell cytotoxicity to self-cells, inhibitory KIRs and NKG2A are also essential for NK cell education, which is a dynamic functional maturation process where constitutive binding of inhibitory receptors to cognate HLA class I molecules (i.e., KIR2DL1/HLA-C2, KIR2DL2-DL3/HLA-C1, KIR3DL1/HLA-Bw4, and CD94-NKG2A/HLA-E) is required for maintaining the full cytotoxic capacity of NK cells (5, 6). The potency of an NK cell is dictated by the strength of continuous interactions via their inhibitory receptor and HLA class I molecules in the surrounding. This process is referred to as tuning (7). As *KIR* and *HLA* segregate independently it is possible for an individual's NK cells to be educated or non-educated by different KIRs.

Although NK cells have been implicated in several autoimmune diseases, their exact role have so far not been established (8). Patients with systemic lupus erythematosus (SLE) have a numerical deficit and a reduced cytotoxicity of NK cells in peripheral blood (9–12). Furthermore, NK cells from SLE patients with active disease have a reduced surface expression of KIR2DL1/2DS1 together with an increased expression of CD94/NKG2A and CD94/NKG2C (12). Genetically, certain KIRs or combinations of KIRs and HLA class I-ligands are associated with increased susceptibility to SLE (13–18). Recently, we demonstrated that a subset (3.4%) of SLE patients harbors functional autoantibodies to the CD94/NKG2A and CD94/NKG2C receptors, which interfere with HLA class I-mediated regulation of NK cell cytotoxicity resulting in a dysregulation of the discrimination between self and non-self-cells (19, 20). To further investigate how common autoantibodies to receptors regulating NK cell cytotoxicity are in systemic autoimmune diseases, we performed a comprehensive screening for autoantibodies targeting eight different KIRs in patients with SLE, primary Sjögren's syndrome (pSS), and systemic sclerosis (SSc). The function of such antibodies was analyzed and their presence was correlated with clinical manifestations.

## Patients and Methods

### Patients and Healthy Controls

Retrospective cohorts of frozen (−80°C) sera from 191 patients fulfilling the 1982 American College of Rheumathology (ACR) classification criteria for SLE (21), 119 patients fulfilling both the 2002 American-European Consensus Group, and 2016 ACR/EULAR criteria for pSS (22, 23), and 48 patients fulfilling the ACR criteria for SSc (24) were included in the study. Sera from 100 healthy donors (HD; Uppsala Bioresource, Uppsala, Sweden) (25) age and sex-matched to the SLE patients were included as controls (Table 1). Clinical data were extracted from medical records. Disease activity of SLE patients at serum sampling was determined using the SLE Disease Activity Index 2000 (SLEDAI-2K) (26). Autoantibody profiles from the SSc patients were determined as previously described (27). The study was approved by the local ethics committee at Uppsala University and Karolinska Institutet (Dnr 013/2009, 399/2000, 024/2007, 217/2006, and 2006/229-31/3) and informed consent was obtained from all patients and controls.

**Table 1:** Baseline characteristics of patients and healthy donors analyzed for anti-KIR autoantibodies.

	**SLE**	**pSS**	**SSc**	**HD**
Number of individuals	191	119	48	100
Female, %	89	92	79	90
Age at serum sampling, mean ± SD years	45 ± 16	55 ± 14	59 ± 15	45 ± 13
ANA, %	98	82	75	
Anti-dsDNA, %	63	2^†^	2	
Anti-Sm, %	30	2^‡^	4	
Anti-RNP, %	29	3^‡^	15	
Anti-SSA/Ro, %	51	78	10	
Anti-SSB/La, %	25	40^§^	2	
Anti-Scl-70, %	1^§^	n.d.	17	

Data are missing from

§ *1*,

† 6, and

‡ *8 patients, respectively*.

*n.d. = not determined*.

### Cells

HEK293T cell lines stably transfected with KIR2DL1 or KIR2DL4 were kindly provided by Professor Eric Long (NIH, Rockville, USA). cDNA encoding KIR2DL2, KIR2DL3, KIR2DS2, KIR2DS4, KIR3DL1, and KIR3DL2 were cloned into a pBABE vector. Plasmids were transfected into HEK293T cells using SuperFect transfection reagent (Qiagen) and surface expression of KIRs determined using flow cytometry. Transfectants with high KIR expression were selected by limiting dilution or FACS sorting (BD FACSAria, BD Biosciences). Peripheral blood mononuclear cells (PBMCs) were isolated using Ficoll density gradient centrifugation and viability frozen in fetal calf serum (FCS) supplemented with 10% DMSO (Sigma) in liquid nitrogen. NK cells were isolated from PBMCs by negative depletion using magnetic-assisted cell sorting (NK cell isolation kit, Miltenyi).

KIR transfectants were cultured in Iscove's Modified Dulbecco's Medium with 1 μg/ml puromycin. Primary cells and K562 cells were cultured in RPMI1640 medium. Both media were supplemented with 10% FCS, 20 mM HEPES, 2 mM Glutamine, 60 μg/ml penicillin, and 100 μg/ml streptomycin (Invitrogen).

### Flow Cytometry Reagents

Fluorescently labeled antibodies to CD56 (NCAM16.2, BD Biosciences), CD3 (SK7 or UCHT1, BD Biosciences), KIR2DL1/2DS1 (EB6B, Beckman Coulter), KIR2DL2/2DL3/2DS2 (GL183, Beckman Coulter or DX27, Biolegend), KIR2DS4 (FES172, Beckman Coulter), KIR2DL4 (mAb33, Biolegend), KIR3DL1 (DX9, BD Biosciences), KIR3DL2 (539304, R&D Systems), and NKG2A (Z199, Beckman Coulter) were used. Dead cells were excluded from the analysis using LIVE/DEAD Fixable Dead Cell Stain Kit (Invitrogen). Binding of human IgG to transfectants was determined using PE-labeled anti-human IgG F(ab)2 donkey fragments (Jackson ImmunoResearch).

### Flow Cytometry

Flow cytometry data were acquired on a FACSCanto II or a LSR II Fortessa instrument (BD Biosciences). Data was compensated and analyzed using FlowJo (TreeStar).

### Detection of Anti-KIR Autoantibodies in Serum

KIR transfectants and the parental untransfected cell line were fluorescently barcoded (28) using CellTraceViolet (Life Technologies) and eFluor670 (eBiosciences) proliferation dye reagents (Supplementary Figure S1). The transfectants were pooled and incubated with human FcBlock (1:100 dilution, Miltenyi) for 10 min on ice to reduce non-specific binding of antibodies. A total of 225,000 cells were incubated with 3.3% serum in 1 × PBS with 10% FCS in 96-well-plates at 4°C for 30 min. Cells were washed twice in 1 × PBS with 2 mM EDTA and 0.5% human serum albumin followed by staining with secondary antibody. Binding of human IgG to cells was determined using flow cytometry. For each serum, the median fluorescent intensity (MFI) signal for each transfectant was divided by the MFI signal of the untransfected cells to yield a specific IgG-binding ratio. The specific IgG-binding ratio was transformed to z-scores [(X–mean_healthy_)/SD_healthy_] and a threshold for antibody positivity was set to 4.

### IFN-α Immunoassay

Levels of IFN-α in serum was determined with an in-house dissociation-lanthanide fluoroimmunoassay (DELFIA) in duplicates, as previously described (29). Briefly, the anti-IFN-α monoclonal antibodies (mAb) LT27:273 and LT27:297 were used as capture antibodies and europium-labeled LT27:297 anti-IFN-α mAb as detection antibody. The detection limit of this assay was 1 U/ml, and sera with a concentration below the limit of detection were assigned a value of 0 U/ml.

### NK Cell Degranulation and Cytotoxicity Assays

SLE IgG was purified from serum or plasma using Protein G GraviTrap columns (GE Healthcare). Human intravenous immunoglobulin (IVIG, Gammagard, Baxter International Inc.) was used as control. For functional studies, PBMCs from HD expressing KIR2DL1, KIR2DL2/DL3, KIR3DL1, and their cognate HLA class I ligands were selected. NK cell activation, as measured by degranulation, and/or fratricide (i.e., self-killing) following exposure to SLE IgG was addressed by incubating overnight IL-2-activated (1,000 IU/ml, Peprotech) PBMCs with or without 20 mg/ml IgG for 2 h. NK cell degranulation was determined by CD107a staining (H4A3, Biolegend), first described by Alter et al. (30), and NK cell viability by Annexin V staining (BD Biosciences). To study detuning, isolated NK cells were pre-incubated with 20 mg/ml IgG for 20 h. NK cell cytotoxicity was measured by a modified Calcein-AM assay (31). Briefly, K562 target cells were labeled with 1 μM Calcein-AM (Sigma Aldrich) at a cell density of 1 × 10^6^ cells/ml for 30 min and then washed in PBS prior to co-culturing with NK cells in triplicates. After 4 h, the cells were spun down and the supernatant discarded. The remaining cells were resuspended in PBS and the fluorescence (535 nm) was measured using a Tecan Infinite F200Pro instrument. Cytotoxicity (target cell death) was calculated based on a maximum fluorescent value (target cells that had not been in contact with effector cells) and a background value (only PBS) using the formula: Cytotoxicity = 1–[(fluorescence of remaining cells–background)/(maximum value–background)]. E:T ratios for the degranulation and the Calcein-AM-based cytotoxicity assay were 1:1 and 10:1, respectively. To study retuning, IgG were washed out and degranulation in response to K562 cells was determined at 48 and 96 h from wash-out.

### Typing and Imputation of KIR and HLA Genotypes

KIR and HLA genotypes were determined using the SSP KIR and KIR-ligand kit (Olerup) (SLE patients and HD in Figure 3) or via imputation from Immunochip (Illumina) genotype data using KIR^*^IMP v1.1.0 (32) and HIBAG v1.18.1 (33), respectively (HD in Figure 4).

### Statistical Analysis

Data were analyzed using Microsoft Excel, Graph Pad Prism v6.02 or R v3.5.1 software. Two-sided Fisher's exact test, Mann Whitney U test, Kruskal-Wallis test and repeated-measure one-way ANOVA were used as specified to assess differences between groups.

## Results

### Autoantibodies Targeting KIRs Are Present in Sera From Patients With SLE, pSS and SSc

**Figure 1:**
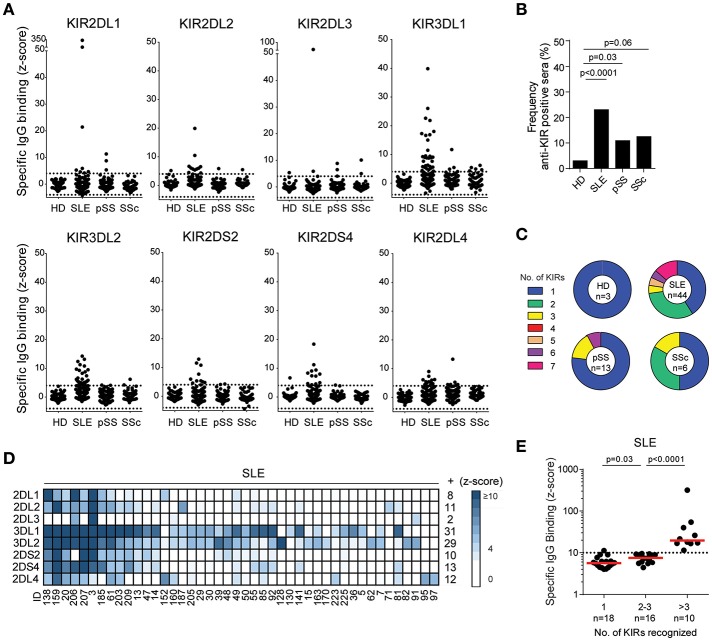
Autoantibodies to KIRs are present in sera from patients with systemic lupus erythematosus, primary Sjögren's syndrome and systemic sclerosis. **(A)** IgG-binding to KIR transfectants in sera from 100 healthy donors (HD), 191 systemic lupus erythematosus (SLE), 119 primary Sjögren's syndrome (pSS), and 48 systemic sclerosis (SSc) patients. Dotted lines denote a z-score of ±4. **(B)** Frequency of HD and patients displaying anti-KIR reactivity to at least 1 KIR. **(C)** The number of KIRs recognized by the anti-KIR-positive sera. **(D)** A heatmap visualizing the KIR-specificity in individual SLE patients. Each column represent serum from one patient and each row one KIR. The number of sera reacting with each KIR is denoted to the right. **(E)** The strongest KIR-reactivity in each sera in SLE patients reacting with 1, 2, 3, or >3 KIRs. Dotted line indicates a z-score of 10. Differences between groups were assessed using the **(B)** Fischer's exact test or **(E)** Mann-Whitney *U*-test.

Initially we screened SLE, pSS, SSc, and HD sera (Table 1) for the presence of IgG that bound to KIR2DL1, 2DL2, 2DL3, 3DL1, 3DL2, 2DS2, 2DS4, or 2DL4-expressing HEK293T transfectants. Autoantibodies to at least one of the KIRs studied were identified in 44 patients with SLE (23.0%), 13 patients with pSS (10.9%), 6 patients with SSc (12.5%), and 3 HD (3.0%) (Figures 1A,B). The frequency of anti-KIR-positive sera was significantly higher in SLE and pSS patients compared to HD controls (*p* < 0.0001 and *p* = 0.03, respectively). Reactivity to each of the eight KIRs was observed in sera from SLE and pSS patients, whereas sera from SSc patients reacted with 4 of the KIRs (Figure 1A). The number of KIRs that each anti-KIR-positive sera reacted with ranged from 1 to 7 (Figures 1C,D). For SLE patients, 59% of the anti-KIR positive sera reacted with ≥2 KIRs and 23% bound to >3 KIRs (Figures 1C,D). In contrast, the majority of anti-KIR-positive sera from HD and patients with pSS and SSc displayed mono-reactivity (Figure 1C). The highest levels of anti-KIR autoantibodies were found in sera from SLE patients (Figure 1A) and increased levels were associated with an increased number of KIRs recognized (Figure 1E). Of the 11 SLE patients with a KIR-binding z-score of >10 to at least one KIR, 10 displayed reactivity to >3 KIRs. Among SLE patients, autoantibodies to KIR3DL1 and KIR3DL2 were most frequent (70.5 and 65.9% of the anti-KIR-positive patients, respectively; Figures 1A,D). Studies of longitudinally sampled sera from 16 SLE patients revealed that the presence and levels of anti-KIR autoantibodies were relatively stable over time (Supplementary Figure S2), but some patients acquired new anti-KIR-specificities and some patients lost KIR-reactivity over time.

In summary, antibodies to KIRs are present in sera from patients with SLE, pSS and SSc. The highest frequency, as well as antibody levels, was found in SLE patients and therefore we focused our further studies on SLE patients.

### Sera From Anti-KIR-positive SLE Patients Block the Binding of Monoclonal Anti-KIR Antibodies to Primary Human NK Cells

**Figure 2:**
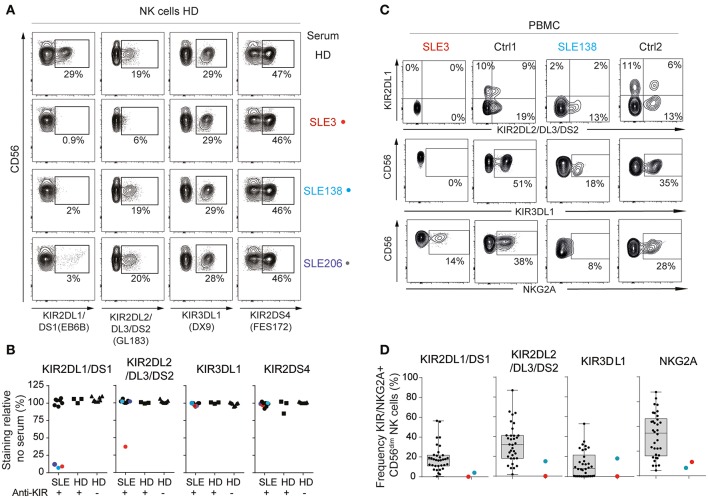
Anti-KIR autoantibodies from SLE patients block the binding of monoclonal anti-KIR antibodies. **(A,B)** Flow-cytometric KIR stainings of healthy donor (HD) cells following 30 min incubation with 50% serum from patients harboring >3 anti-KIRs (*n* = 10), KIR-positive HD (*n* = 3), and KIR-negative (*n* = 8) HD **(A)** Flow cytometry plots from one representative HD and the three SLE patients that blocked the binding of anti-KIR2DL1/DS1. **(B)** The frequency of KIR-positive cells in serum-treated cells relative to the mean frequency of KIR-positive cells in eight non-treated cells. **(C,D)** Flow-cytometric KIR stainings of CD3^−^CD56^dim^ NK cells in PBMCs from anti-KIR-negative (*n* = 34), and anti-KIR-positive SLE patients (*n* = 2). Data are from two separate experiments. **(C)** Flow cytometry plots from SLE3, SLE138 and one representative HD from each experiment. **(D)** Data for all patients presented as boxplots, with the median, interquartile range, and range denoted.

In an attempt to confirm the presence of anti-KIR autoantibodies and to address their specificity in more detail, sera from the 10 SLE patients reacting with >3 KIRs, the three anti-KIR-positive HD, and eight anti-KIR-negative HD were analyzed for their ability to interfere with the binding of monoclonal anti-KIR antibodies to primary NK cells from a HD. Sera from 3 of the 10 SLE patients with KIR reactivity to >3 KIRs blocked the binding of the anti-KIR2DL1/2DS1 mAb EB6B (SLE3, SLE138, and SLE206; Figures 2A,B). Notably, these three sera had the highest anti-KIR2DL1 levels (Figure 1D). Serum from patient SLE3 was the only serum that bound KIR2DL2, KIR2DL3, and KIR2DS2 (Figure 1D). Accordingly, this was the only serum that blocked anti-KIR2DL2/DL3/DS2-binding (clone GL183). In contrast, serum taken from patient SLE3 at an earlier time-point, where no or low levels of antibodies to KIR2DL1/DL2/DL3/DS2 were present, did not block the binding of these two antibodies (data not shown). None of the anti-KIR-positive sera from SLE patients blocked the binding of anti-KIR3DL1 (clone DX9) or anti-KIR2DS4 (clone FES172). In terms of anti-KIR-positive HD, the serum that reacted with KIR2DS4 interfered slightly with anti-KIR2DS4-binding, whereas none of the other anti-KIR-positive or anti-KIR-negative HD sera affected anti-KIR-binding (Figure 2B).

Flow cytometric stainings of PBMCs from SLE3 and SLE138 revealed that NK cells from SLE3 lacked detectable expression of cell surface KIR2DL1, KIR2DL2/DL3/DS2, and KIR3DL1, but had detectable surface expression of NKG2A (Figures 2C,D). NK cells from SLE138 lacked surface expression of KIR2DL1 but had low cell surface expression of KIR2DL2/DL3/DS2 and NKG2A (Figures 2C,D).

Together, these data confirm the presence and specificity of anti-KIR autoantibodies and demonstrate that NK cells from patients with anti-KIR autoantibodies have an altered KIR phenotype.

### Anti-KIR Autoantibodies Detune NK Cells

**Figure 3:**
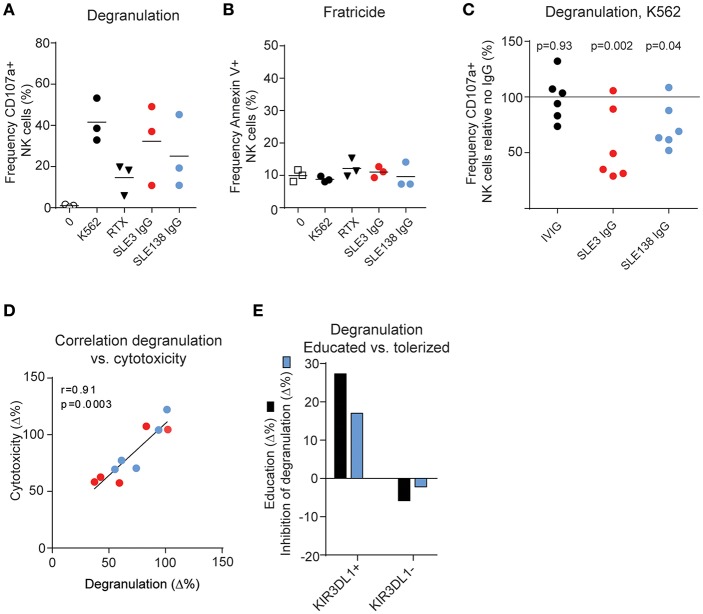
Anti-KIR autoantibodies induce hyporesponsiveness in NK cells. **(A)** NK cell degranulation and **(B)** viability of IL-2-activated PBMCs from 3 HDs following incubation with K562 cells, rituximab (RTX, anti-CD20), or IgG as indicated. **(C)** K562-induced degranulation in NK cells from 6 HDs exposed to IgG from patient SLE3, SLE138, or HDs (IVIG) relative to no IgG. *P*-values from a repeated measures one-way ANOVA comparing the relative degranulation in IgG-treated cells to untreated cells are shown. **(D)** Correlation between the difference in degranulation and the difference in cytotoxicity of IgG-treated NK cells compared to non-treated NK cells. **(E)** Comparison of the education level and the inhibitory effect of SLE138-IgG on degranulation compared to no IgG. Education level was defined as the difference in degranulation in indicated NK cell subsets relative to KIR-negative NK cells (KIR2DL1/DS1^−^KIR2DL2/DL3/DS2^−^KIR3DL1/DS1^−^) in the absence of IgG. **(A–E)** Data from patient SLE3 and SLE138 are colored in red and blue, respectively.

To study the functional effects of anti-KIR autoantibodies we isolated IgG from two of the SLE patients whose sera blocked binding of the monoclonal anti-KIR antibodies. First, we addressed whether IgG containing anti-KIR autoantibodies triggered NK cell activation and/or apoptosis of KIR-expressing NK cells. Exposure of PBMCs from HDs to IgG rapidly triggered strong NK cell activation as determined by NK cell degranulation (Figure 3A). However, this was not accompanied by an increased NK cell death (Figure 3B).

**Figure 4:**
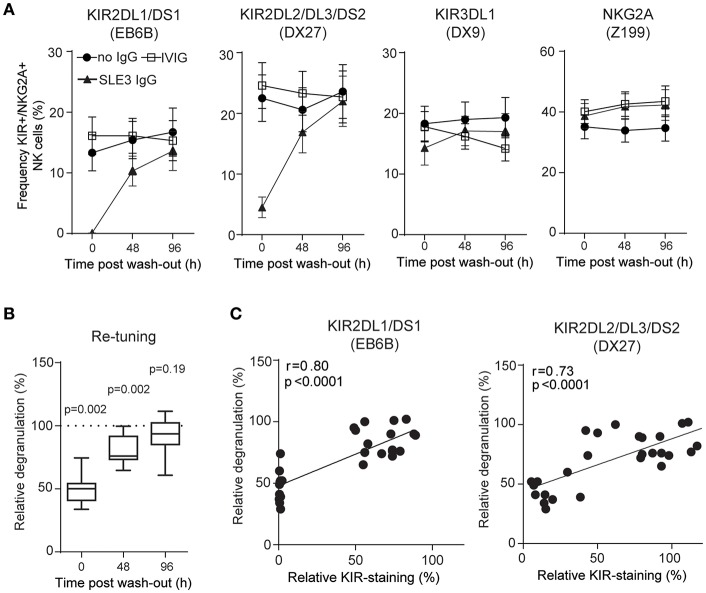
NK cells retune following wash-out of IgG. **(A)** Surface stainings of indicated NK cell receptors, and **(B)** degranulation of NK cells from 10 HDs was determined before (0 h), and after wash-out of IgG (48 and 96 h). Degranulation of NK cells treated with IgG from SLE3 relative to IVIG-treated cells. Data are shown as **(A)** mean values with error bars representing the standard error of the mean, and **(B)** boxplots with median, interquartile range, and range. **(B)** The one-sample *t*-test was used to assess whether the relative degranulation differed from 100%. **(C)** Correlation between data in **(A,B)**. The *p*-values and Pearson's correlation coefficients (*r*) from linear regressions are denoted.

KIR and KIR-ligand genotyping of DNA from patient SLE3 and SLE138 revealed the presence of *KIR2DL1, KIR2DL2, KIR2DL3*, and *KIR3DL1*, as well as genes for KIR-ligands containing the HLA-C1, HLA-C2, and Bw4 epitopes, indicating that they were educated on these KIRs (Supplementary Table S1). To study whether anti-KIR autoantibodies interfered with NK cell education, we exposed isolated HD NK cells over night with IgG and measured the degranulation toward HLA class I-negative K562 cells, used as the gold standard NK cell target cell line. Exposure of NK cells to IgG from patient SLE3 and SLE138 resulted in a reduced degranulation capacity (*p* = 0.002 and *p* = 0.04, respectively; Figure 3C) and the decreased degranulation correlated with a reduced killing of K562 cells (Figure 3D). Since anti-KIR-containing IgG blocked the detection of KIR molecules it was not possible to study the effect on degranulation in single-positive KIR subsets of NK cells. However, NK cells from one HD were educated on KIR3DL1 (i.e., increased degranulation compared to KIR-negative NK cells in the absence of IgG), but tolerized in the KIR3DL1-negative NK cell subset (i.e., decreased degranulation compared to KIR-negative NK cells in the absence of IgG) (34). Consistent with an abrogated NK cell education, IgG from SLE138 reduced the degranulation level by the educated KIR3DL1-positive NK cell subset while increasing the degranulation levels within the tolerized KIR3DL1-negative NK cell subset (Figure 3E). IgG from patient SLE3 partially blocked KIR3DL1 detection in this donor, which precluded the same analysis for this patient. In line with the dynamic process of NK cell education, the effects of SLE-IgG on degranulation was reversible. Prolonged incubation of NK cells after wash-out of anti-KIR autoantibodies resulted in the re-appearance of detectable KIR molecules on the NK cell surface (Figure 4A), concomitant with an increased degranulation toward K562 cells (Figures 4B,C). Both patient SLE3 and SLE138 carried the A/B KIR-haplotype (Supplementary Table S1), which contain activating KIRs. Importantly, IgG from patient SLE3 detuned NK cells to the same extent in HDs carrying the A/A as those carrying the A/B KIR haplotype (*p* = 0.32; data not shown).

Together, these data show that anti-KIR autoantibodies found in patients with SLE induce hyporesponsiveness of NK cells and that NK cell function returns when KIR expression reappears.

### The Presence of Multiple Anti-KIR Autoantibodies Is Associated With an Increased Disease Activity and Elevated Serum Levels of IFN-α

**Figure 5:**
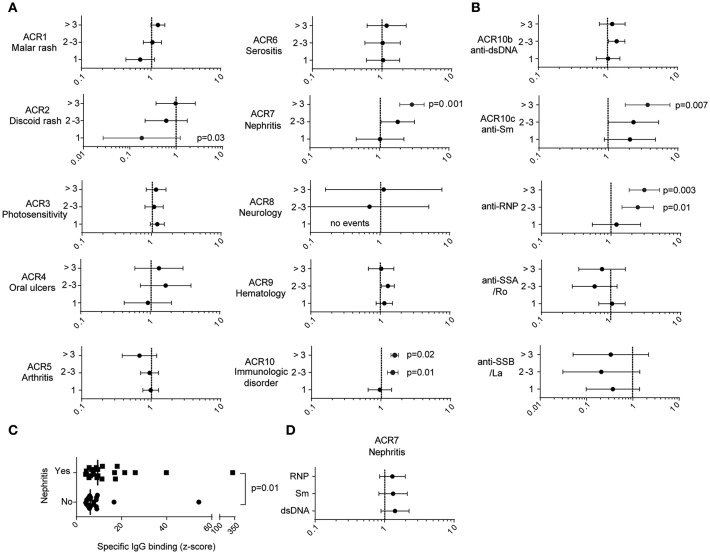
The presence of anti-KIR autoantibodies to >3 KIRs is associated with an increased risk for nephritis and the presence of anti-Sm antibodies. **(A,B,D)** Forest plots depicting the relative risk and 95% confidence intervals of fulfilling clinical characteristics as specified. **(A,B)** The relative risk of SLE patients presenting with 1, 2, 3, or >3 anti-KIR autoantibodies compared to anti-KIR-negative patients. **(C)** The strongest levels of anti-KIR autoantibodies in anti-KIR-positive SLE patients stratified by the presence (*n* = 21) or absence (*n* = 23) of nephritis. The median values are denoted with vertical bars. **(D)** The relative risk of fulfilling the ACR criteria for nephritis in patients positive relative to negative for the indicated autoantibodies. **(A,B,D)** Fischer's exact test with *p* < 0.05 denoted in the graphs. **(C)** Mann-Whitney *U*-test.

To address whether the presence of anti-KIR autoantibodies is associated with any clinical or laboratory parameters, we reviewed the medical records, analyzed serum IFN-α levels and compared them between SLE patients harboring 0, 1, 2, 3, or >3 anti-KIR autoantibodies. The four groups had a similar age and disease duration at the time-point when anti-KIR autoantibodies were analyzed (Table 2). Patients with anti-KIR autoantibodies targeting >3 KIRs had a significantly higher number of ACR criteria fulfilled (median 7 vs. 6, *p* = 0.04), a higher disease activity (median 13 vs. 2, *p* < 0.0001) and increased levels of IFN-α in serum (median 11.6 U/ml vs. 0 U/ml, *p* < 0.0001) compared with anti-KIR-negative patients. Whereas, no significant differences in organ damage or treatment were observed (Table 2). When analyzing each ACR criterion separately we found that patients with >3 anti-KIR autoantibodies had an increased risk of fulfilling the criteria for nephritis (80.0 vs. 27.2%, *p* = 0.001) and immunologic disorder (100 vs. 63.3%, *p* = 0.02) compared to patients with no anti-KIR autoantibodies (Figure 5A). The association to nephritis was not specific to any subtype of nephritis (Supplementary Table S2). Subdivision of the immunologic ACR criteria revealed that the presence of antibodies to >3 KIRs was associated with anti-Sm (50.0 vs. 13.6%, *p* = 0.007), but not with anti-dsDNA antibodies (70.0 vs. 60.5%, *p* = 0.74) (Figure 5B). In terms of other autoantibodies, the presence of >3 anti-KIR autoantibodies was associated with anti-RNP antibodies (70.0 vs. 23.1%, *p* = 0.003), but not anti-SSA/Ro (*p* = 0.52), or anti-SSB/La (*p* = 0.28). Consistent with the positive correlation between anti-KIR levels and number of KIRs recognized (Figure 1E), the levels of anti-KIR autoantibodies were higher in patients with nephritis compared to those without nephritis (*p* = 0.01; Figure 5C). Importantly, despite the association of >3 anti-KIR-autoantibodies with anti-Sm and anti-RNP, the increased presence of nephritis in these patients was not driven by an association of anti-Sm or anti-RNP (*p* = 0.32 and *p* = 0.30, respectively; Figure 5D).

**Table 2:** Clinical characteristics of SLE patients stratified by their number of anti-KIR autoantibodies.

**Number of anti-KIRs**	**0**	**1**	**2-3**	**>3**	***p*^†^**
Number of individuals	147	18	16	10	
Age at disease-onset, Years	30 (20–40)	30 (19–42)	27 (19–40)	21 (16–40)	0.74
Age at serum sampling, Years	45 (30–58)	43 (30–50)	47 (34–61)	32 (22–52)	0.42
Disease duration^*^, Years	16 (11–29)	20 (11–39)	18 (9–28)	11 (3–25)	0.54
Number of ACR criteria	6 (5–7)	6 (4–6)	7 (5–7)	7 (7–8) ^‡^	0.02
SLEDAI-2K	2 (0–4)	2 (1–7)	2 (1–7)	13 (7–15)^§^	<0.0001
SDI	1 (0–3)	0 (0–2)	1.5 (0–3)	1.5 (0–4)	0.31
Serum IFN-α, U/ml	0 (0–0)	0 (0–1.1)	0 (0–3.7)	11.6 (5.2–35.0)^§^	<0.0001
Treatment					
Glucocorticoids, %	57.9	55.6	73.3	90.0	0.15
Hydroxychloroquine/ Chloroquine, %	51.0	50.0	46.7	40.0	0.93
Azathioprine, %	23.4	27.8	26.7	0	0.31
Mycophenolate mofetil, %	7.6	5.6	26.7^‡^	20.0	0.05
Methotrexate, %	6.2	0	0	10.0	0.51

*Data are presented as median (interquartile range) or as frequencies (%). SLEDAI-2K = SLE Disease Activity Index 2000, SDI = Systemic Lupus International Collaborating Clinics/American College of Rheumatology Damage Index*.

* *At medical record review*.

† *The Kruskal-Wallis test (numerical values) or the Fischer's exact test (categorical values) was used to compare all 4 groups. For p <0.05 each of the three anti-KIR positive groups were compared to the anti-KIR-negative group using the Dunn's multiple comparisons test or the Fischer's exact test and p < 0.05 are indicated*.

‡ *p = 0.04*;

§ *p < 0.0001*.

*Data for serum IFN-α are from 123 (0 anti-KIR), 15 (1 anti-KIR), 12 (2-3 anti-KIR), and 8 patients (>3 anti-KIR). Data for SLEDAI-2K are from 138 (0 anti-KIR), 17 (1 anti-KIR), 13 (2-3 anti-KIR), and 10 patients (>3 anti-KIR)*.

Thus, autoantibodies to multiple KIRs are mainly found in SLE patients presenting with high disease activity, increased serum levels of IFN-α and nephritis.

## Discussion

In this study we performed a screening for autoantibodies to eight different KIRs in sera of patients with SLE, pSS, and SSc. Twenty-three percent of the SLE patients displayed IgG-reactivity to at least one KIR. These data are line with a previous study, which described antibodies reacting with KIR2DL1 or KIR2DL3-receptors in 7 of 30 (23.3%) SLE patients by probing SLE sera for reactivity to *E. coli*-derived recombinant KIR2DL1 and KIR2DL3 in Western blot (35). A recent study demonstrated autoantibodies to KIR3DL1 in 22 of 28 SLE sera analyzed with ELISA using recombinant KIR3DL1 (36). In addition to confirming the presence of anti-KIR autoantibodies in SLE patients, our data demonstrate that these autoantibodies react with natively folded membrane-bound KIRs expressed on human cells, and that reactivity to KIR2DL2, KIR3DL2, KIR2DS2, KIR2DS4, and KIR2DL4 is also present in SLE patients. Furthermore, we describe anti-KIR autoantibodies in pSS and SSc patients, albeit at lower frequencies than in SLE patients (10.9 and 12.5%, respectively). In this paper we also link SLE IgG-mediated blockade of KIR on HD NK cells to a decrease in cytotoxicity, which may explain the poor NK cell function in patients with SLE (9–11).

The highest levels of anti-KIR autoantibodies were found in SLE patients, and increased levels were associated with a broader KIR reactivity. Autoantibodies reacting with KIR3D receptors were more prevalent, and their levels generally higher, than autoantibodies reacting with KIR2D receptors. The reason for this is unclear, but it may relate to the increased size of the molecules and thereby increased number of possible autoantigens on KIR3D receptors, or to the mechanisms whereby anti-KIR autoantibodies are produced. Among the pSS and SSc patients, reactivity to >3 KIRs was only observed in 1 pSS serum. The three anti-KIR-positive HD only reacted with 1 KIR each and their levels were low. The presence of anti-KIR autoantibodies at a low frequency in HD are in line with a previous study describing memory B cells reacting with KIRs in HD (37).

Molecular mimicry is one mechanism implicated in the break of tolerance. A recent study identified a penta-peptide (SKVVS) in the human papillomavirus (HPV) protein L1 that is shared with KIR2DL1, 2DL2, 3DL1, 3DL2, and 2DL4 and suggested that HPV infection in genetically predisposed individuals may contribute to lupus via molecular mimicry (38, 39). In this light, it would be interesting to study whether the presence of anti-KIR autoantibodies correlates with HPV infection, but the lack of these data in our cohort preclude such analyses.

Sera from 3 of the 10 patients reacting to >3 KIRs (SLE3, SLE138, and SLE206) blocked the binding of the anti-KIR2DL1/DS1 mAb EB6B, and one of these sera (SLE3) also blocked the binding of the anti-KIR2DL2/DL3/DS2 mAb GL183. Both EB6B and GL183 have previously been shown to interfere with KIR-mediated NK cell inhibition (40). Despite the fact that KIR-genotyping demonstrated the presence of *KIR2DL1, KIR2DL2*, and *KIR2DL3* in these patients, NK cells from SLE3 and SLE138 did not stain, or had a very low level of KIR2DL1/DS1, and KIR2DL2/DL3/DS2 staining was absent from SLE3. A possible explanation for these findings is that anti-KIR autoantibodies bound KIRs *in vivo* and masked the detection of the flow antibody, however it cannot be ruled out that KIR2DL1/DS1 and/or KIR2DL2/DL3/DS2-expressing NK cells in these patients had been depleted or by other means removed from the circulation.

Lirilumab is a pan-KIR2D mAb that have been evaluated in clinical trials with the rationale of augmenting NK cell cytotoxicity toward cancer cells by blocking inhibitory KIRs (41). However, clinical trials have not met their end-points (42). Mechanistic studies suggest that the lack of efficacy may be due to a decreased NK cell cytotoxicity because of anti-KIR2D-antibody-mediated detuning of KIR2D-educated NK cells (43). Consistent with an effect on NK cell education, treatment of NK cells with IgG from two of the anti-KIR-positive patients in this study induced detuning of NK cell function as measured by decreased degranulation toward K562 cells. Following wash-out of IgG, KIR surface expression gradually recovered concurrent with the recovery of NK cell function. These data suggest that antibody-mediated blockade of KIRs and the detuning of NK cells may be one mechanism contributing to the decreased cytotoxicity previously reported in NK cells from lupus patients (9–11). The detuning of NK cells may also contribute to the fact that the cytotoxicity of NK cells from lupus patients with poor basal NK cell function is not enhanced by type I interferon (44). Given that decreased killing of activated T cells by NK cells has previously been proposed as a mechanism for the development of autoimmunity in rheumatoid arthritis and multiple sclerosis (45, 46), one could speculate that such mechanisms may also operate in SLE.

The presence of autoantibodies reacting with >3 KIRs was associated with a higher disease activity, elevated levels of IFN-α, and an increased risk for fulfilling the nephritis and the immunologic ACR criteria. The immunologic criterion includes having anti-dsDNA and/or anti-Sm autoantibodies, both of which presence have been associated with lupus nephritis previously (47). In our study, the presence of autoantibodies reacting with >3 KIRs was associated with anti-Sm and anti-RNP antibodies, but not anti-dsDNA antibodies. Thus, these patients resemble the subgroup of SLE patients with high IFN signature, anti-Sm/RNP antibodies and nephritis previously described by Kirou et al. (48). Importantly, the association with anti-KIR autoantibodies and an increased frequency of nephritis, were not driven by the presence of anti-Sm-autoantibodies as these were not significantly associated with nephritis in our relatively small cohort. It is notable that in our previous study, the presence of autoantibodies to the CD94/NKG2A receptor was also associated with an increased risk for nephritis (20). Thus, autoantibodies targeting inhibitory NK cell receptors may be a common phenomenon in patients with lupus nephritis. Regarding the pSS and SSc patients, the number of anti-KIR-positive patients was too few to perform analysis of clinical association. However, it is worth noting that the only pSS patient presenting with >3 KIR autoantibodies had interstitial nephritis, a manifestation that only 1 out of 106 of the anti-KIR-negative pSS patients displayed. At this point we do not know whether anti-KIR autoantibodies have a causative role in the development of nephritis or whether they represent an epiphenomenon, but it is interesting to note that a study of 512 patients with end-stage renal disease (ESRD) due to chronic glomerulonephritis, chronic interstitial nephritis, hypertensive nephrosclerosis or diabetic nephropathy and 512 healthy controls found a larger proportion of individuals with less educated NK cells in patients with ESRD (i.e., negative for either KIR2DL1/HLA-C2, KIR2DL2/HLA-C1, or KIR3DL1/HLA-Bw4) (49). Although further studies are warranted to examine the connection between NK cell education and nephritis, this finding raises the possibility that both a genetic and an antibody-mediated interference with NK cell education may contribute to the development of renal disease of both autoimmune and non-autoimmune etiology.

One limitation of this study is that, even though this is the most comprehensive screening for KIR autoantibodies performed so far, we did not screen for autoantibodies to all 15 KIRs. Thus, we may have underestimated the prevalence of patients harboring autoantibodies to KIRs. However, the large homology between the extracellular domains of the inhibitory and corresponding activating KIRs, as well as the large homology among KIRs in general, suggest that this is not likely to have a large impact on the results. Another limitation of the study is that we did not have sufficient IgG to isolate the anti-KIR-specific autoantibodies for detailed functional studies. However, the fact that the effect of anti-KIR-containing IgG was observed on isolated NK cells, that IgG had opposite effects in educated and tolerized subsets of NK cells and that NK cells retuned following wash-out of IgG strongly suggest that the effect on NK cells were at least in part the result of antibody-mediated blockade of KIR/KIR-ligand interactions. This speculation is not unlikely given the clear data on the effect on NK cells that are triggered upon exposure to the anti-KIR2D antibody lirilumab (39).

In conclusion, our study demonstrates that autoantibodies to KIRs are frequently found in SLE patients. The strong association with increased disease activity and nephritis, and their functional effect on NK cell cytotoxicity suggest that anti-KIR autoantibodies may be clinically relevant.

## Data Availability

The datasets generated for this study are available on request to the corresponding author.

## Ethics Statement

The studies involving human participants were reviewed and approved by the local ethics committee at Uppsala University and the local ethics committee at Karolinska Institutet. The patients/participants provided their written informed consent to participate in this study.

## Author Contributions

NH, MC, FS, and CL contributed to the conception and designed the study. NH and FS performed the experiments. DL, SR, and GN provided the patient sera and reviewed medical records. NH, MC, FS, and CL analyzed and interpreted the data. All authors were involved in drafting the article or revising it critically for important intellectual content, and all authors approved the final version to be published.

### Conflict of Interest Statement

The authors declare that the research was conducted in the absence of any commercial or financial relationships that could be construed as a potential conflict of interest.
