# Supplementary material for: Autoantibodies to Killer Cell Immunoglobulin-Like Receptors in Patients With Systemic Lupus Erythematosus Induce Natural Killer Cell Hyporesponsiveness
Source: Front Immunol. 2019 Sep 11;10:2164. doi: 10.3389/fimmu.2019.02164 (PMC6749077; doi:10.3389/fimmu.2019.02164)
Supplement: Supplementary file 1 [file Data_Sheet_1.docx]

**Supplementary Table S1. KIR and KIR-ligand genotype**

| **KIR/HLA** | **SLE3** | **SLE138** |
| --- | --- | --- |
| KIR2DL1 | YES | YES |
| KIR2DL2 | YES | YES |
| KIR2DL3 | YES | YES |
| KIR2DS1 | NO | NO |
| KIR2DS2 | YES | YES |
| KIR2DS3 | NO | NO |
| KIR3DL1 | YES | YES |
| KIR3DS1 | NO | NO |
| HLA-C1 | YES | YES |
| HLA-C2 | YES | YES |
| HLA-Bw4 | YES | YES |

The presence or absence of indicated KIR and KIR-ligand as determined by SSP typing.**Supplementary Table S2. WHO sub-classification of nephritis in patients with anti-KIR autoantibodies reacting with >3 KIRs.**

| **ID** | **No of anti-KIRs** | **WHO classification^1^** |
| --- | --- | --- |
| SLE138 | 7 | VA |
| SLE159 | 7 | IVA |
| SLE20 | 7 | IIIC |
| SLE206 | 7 | V+IVA |
| SLE207 | 7 | IVB |
| SLE185 | 6 | IVC |
| SLE61 | 6 | IIA |
| SLE203 | 5 | VB |

^1^Churg J, Bernstein J, Glassock RJ. Lupus nephritis. In: Renal disease: classification and atlas of glomerular diseases. 2nd ed.New York:Igaku‐Shoin;1995. p.151–5.
