# Supplementary figures and images for: Autoantibodies to Killer Cell Immunoglobulin-Like Receptors in Patients With Systemic Lupus Erythematosus Induce Natural Killer Cell Hyporesponsiveness
Source: Front Immunol. 2019 Sep 11;10:2164. doi: 10.3389/fimmu.2019.02164 (PMC6749077; doi:10.3389/fimmu.2019.02164)

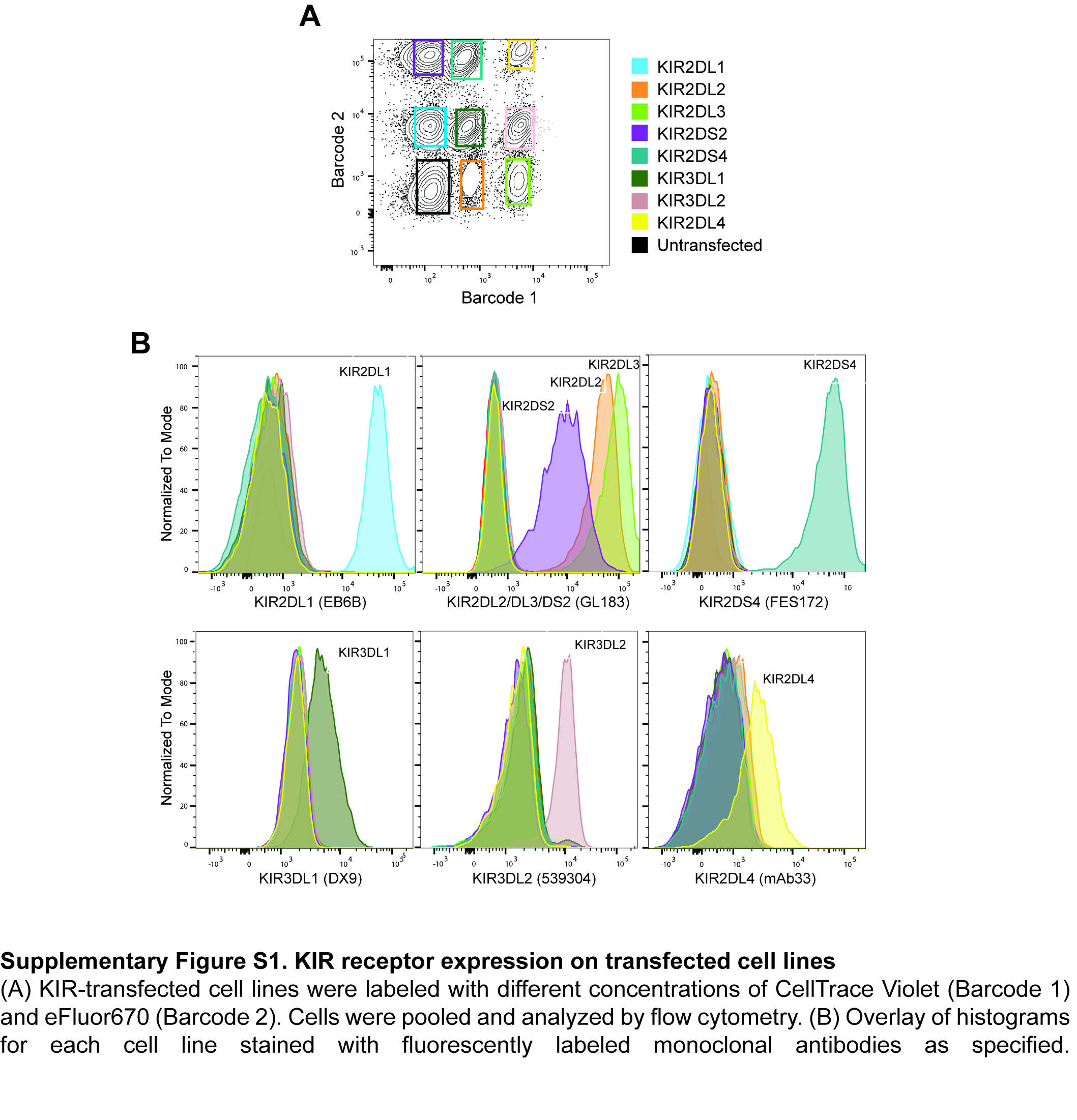

Supplement: Supplementary file 2 [file Image_1.TIF]

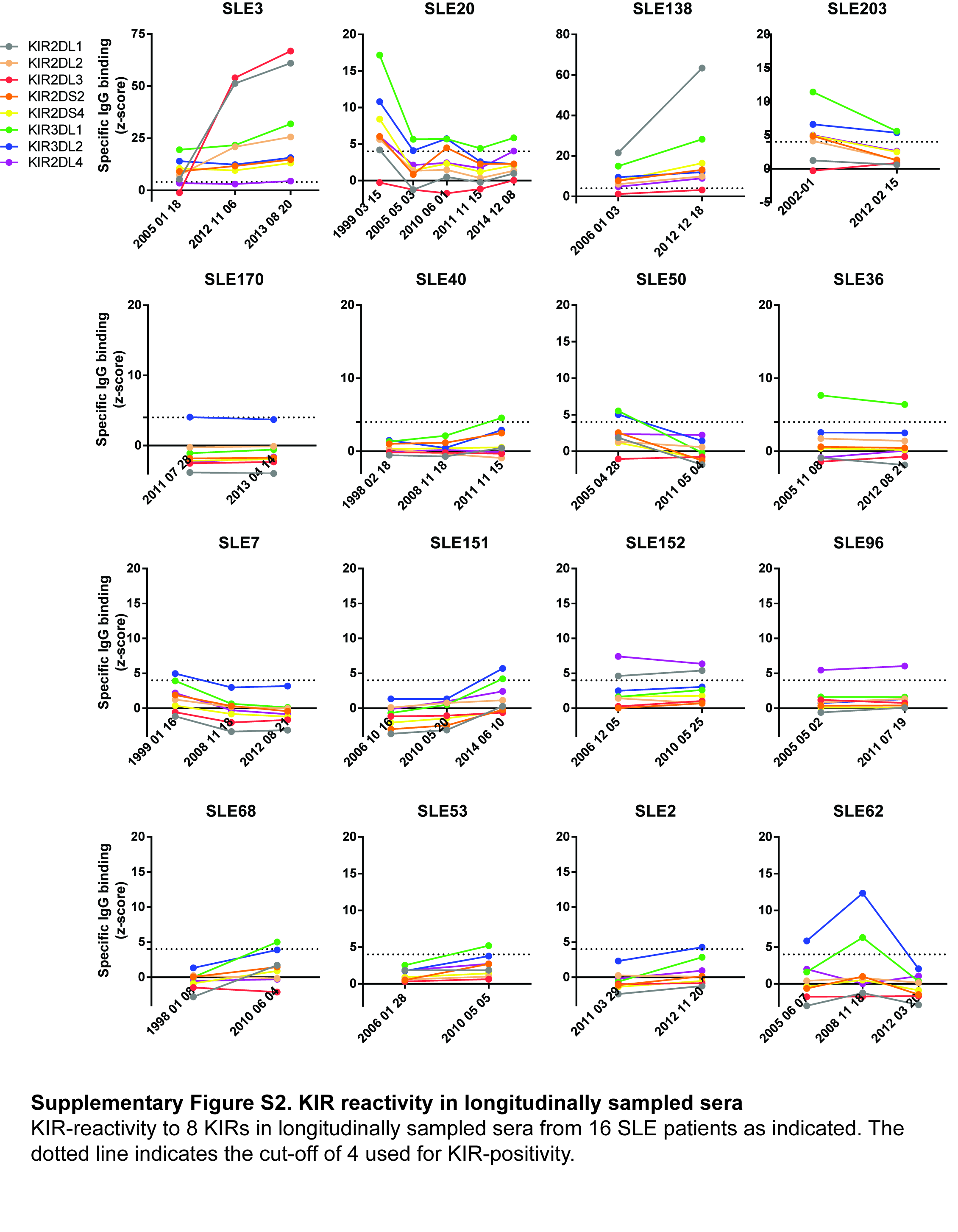

Supplement: Supplementary file 3 [file Image_2.TIF]
